# The relationship between perceived family conflicts and unhealthy lifestyles among adolescents: the interlocking mediating effect of mental health and school adaptation

**DOI:** 10.1186/s12889-025-25747-w

**Published:** 2025-11-28

**Authors:** Yuanhang Zhang, Jin Zhou, Xiaoshuai Zhang, Yan Zhang, Yuzhu Hou, Lin Zhang, Lei Gao

**Affiliations:** School of Health Management, Harbin Medical University, Harbin, Heilongjiang China

**Keywords:** Teenagers, Perception of family conflicts, Unhealthy lifestyle behaviors, Mental health, School adaptation

## Abstract

**Background:**

Globally, issues related to unhealthy lifestyle behaviors among teenagers have become a widely concerned topic. Family conflict perception, as one of the influencing factors of unhealthy lifestyle behaviors among teenagers, has received less attention due to its underlying pathway mechanism. Exploring the interlocking mediating role of mental health and school adaptation between perceived family conflicts and unhealthy lifestyle behaviors can provide new ideas for preventing unhealthy lifestyle behaviors among adolescents.

**Method:**

This study was designed as a cross-sectional study, and the data were derived from the Youth Health Database of the National Population Health Science Data Center (PHDA) in 2020–2021. After data sample selection and cleaning, a total of 9,114 participants were included in the study. The study used t-tests and ANOVA to test for demographic differences. Pearson correlation was used to test the correlation between research variables. The proposed mediation model was tested using the Hayes PROCESS macro in SPSS.

**Results:**

Unhealthy lifestyle behaviors of adolescents were negatively correlated with perceived family conflicts (*r* = -0.27, *p* < 0.01) and school adaptation (*r* = -0.29, *p* < 0.01), and positively correlated with mental health (*r* = 0.30, *p* < 0.01). Mental health and school adaptation play a partial mediating role in perceiving the relationship between family conflicts and unhealthy lifestyle behaviors. A high level of perception of family conflict is not only associated with the occurrence of unhealthy lifestyle behaviors among teenagers, but also has a negative impact on their mental health, thereby hindering the development of teenagers’ adaptation to school and possibly resulting in the emergence of unhealthy lifestyle behaviors.

**Conclusion:**

This study confirmed the chain mediating role of mental health and school adaptation between perceived family conflicts and unhealthy lifestyle behaviors among adolescents. This study enhances our understanding of the connection mechanism between family conflict perception and unhealthy lifestyle behaviors among adolescents. The improvement of teenagers’ mental health and school adaptation level can reduce the possibility of the development of unhealthy lifestyle behaviors caused by the perception of family conflicts. This study provides information for the intervention of schools, social organizations and other entities in promoting the well-being of adolescents and preventing their unhealthy lifestyle behaviors.

## Introduction

Unhealthy lifestyle behaviors refer to behaviors and habits associated with an increased risk of health [1]. Adolescence, as a crucial stage in the transition from childhood to adulthood, is the core period for the formation of lifestyle and behavioral habits [2]. Due to the immaturity of psychological development [3], and the pressure from school, family and society, teenagers are prone to harmful behaviors such as smoking and unhealthy diet [4–6]. A study condcuted in 2021 has found that approximately 90% of Chinese teenagers have two or more unhealthy lifestyles [7], and the proportion of various unhealthy lifestyle behaviors increases with age [8]. In 2019, United Nations Children’s Fund (UNICEF) stated that approximately 70% of premature deaths are related to unhealthy behaviors such as smoking and drinking that begin during adolescence [9]. Therefore, the unhealthy lifestyle behaviors of teenagers have become an important social issue. This is related to personal factors, family characteristics, and social and environmental conditions [10, 11]. Studies have shown that family environmental factors are important social factors influencing health related risk behaviors [12, 13]. Previous studies by scholars have shown that the driving force behind human behavior stems from the undercurrents of family life [14, 15]. The theory of behavioral addiction further indicates that teenagers may cope with tense atmospheres or psychological stress at home by smoking and drinking. Existing research has confirmed the association between family conflicts and unhealthy lifestyle behaviors among adolescents, but there are still limitations: (1) At the mechanism explanation level, related discussions often focused on a single dimension, and the dynamic process and integrated framework of the evolution of family conflicts into health-risk behaviors still need to be explored [16, 17]. (2) At the level of cultural adaptability, most of the current evidence stems from Western individualistic culture. Under the academic pressure from East Asia, the adaptability of Chinese teenagers remains to be verified [8]. (3) Behavioral measurement level, the measurement of unhealthy lifestyle behaviors mainly focuses on the use of substances such as tobacco and alcohol at present [12, 18], while the measurement of emerging behaviors such as excessive use of digital media is still in the exploratory stage and needs further improvement to adapt to the changes in the behavioral characteristics of contemporary teenagers.

Family conflict perception refers to an individual’s subjective feelings and cognition of tense, unstable or conflicting states within a family.This kind of cognition not only encompasses direct conflict events but also involves potential family stressors and so on [19]. Although teenagers spend more time interacting with their peers at school, the family atmosphere still has a significant impact on their emotional and behavioral development. According to the theory of emotional safety, conflicts and tense atmospheres within the family may reduce the emotional safety of teenagers, thereby affecting their mental health and behavioral performance. Existing research indicates that a conflicting family atmosphere is associated with the internalization problems among adolescents [20]. However, a good family environment can prevent drug abuse, sexual risky behaviors, depressive symptoms, etc., especially during pubertal timing [21, 22]. Some studies have also found that despite the differences in gender, family dysfunction may be related to negative emotions such as depression in teenagers, which may prompt them to relieve stress through unhealthy behaviors like drinking [23]. A tense and anxious family atmosphere may also weaken teenagers’ self-control and reduce their ability to recognize and judge dangerous behaviors. A study found even indifferent family atmosphere like parental phubbing may increases their likelihood of engaging in unhealthy lifestyle behaviors [24]. Based on this, we believe that the perception of family conflict is associated with the unhealthy lifestyle behaviors of teenagers.

Mental health issues among teenagers have always been emphasized as an important factor affecting the welfare of teenagers. Relevant studies have shown that family environment is significantly related to the mental health of teenagers. Problematic marriages, including quarrels, verbal abuse and physical conflicts between spouses, may cause a variety of negative reactions among teenagers and reduce mental health [25, 26]. The negative impact of low-quality or high-conflict marital relationships on the mental health of adolescents may manifest as psychological problems, such as internalization and externalization disorders, depression, anxiety and mood disorders [27, 28]. The high school stage is a crucial period for the rapid psychological and physical development of teenagers. High school students have unique psychological development characteristics and are prone to emotional fluctuations and emotional sensitivity [29].Emotional dynamics may affect an individual’s psychological and social functions [30]. Without proper diversion, harmful behaviors such as smoking, drinking and Internet addiction may turn into negative coping styles. Based on this evidence, we believe that mental health is a mediating variable that influences the perception of family conflict and unhealthy lifestyle behaviors.

School adaptation is a combination of cognitive attitudes and behaviors, enabling students to live and study healthily and succeed on campus [31]. Studies have shown that adolescents with a high level of family functional environment are more adapted to school than those with a low level [32, 33]. The higher the frequency and intensity of family conflicts that children and adolescents experience, the more likely they are to develop sleep disorders [34],and they may also encounter other emotional and behavioral adjustment problems.

At the same time, the emotions and behaviors of family members will “overflow” into their interactions with children, thereby affecting children’s adaptation to school [35]. Some studies have also shown that school adaptability is positively correlated with students’ academic performance [36], subjective well-being [37], and social support [38]. On the contrary, teenagers with poor school adaptability are more likely to experience negative events, such as mobile phone addiction [39, 40], school bullying [41], and alcohol abuse [42]. Based on this, we believe that school adaptation plays a mediating role between the perception of family conflicts and unhealthy lifestyle behaviors.

As teenagers grow older, they are confronted with multiple pressures in terms of academic studies, social interactions and future planning. These stress factors not only threaten their mental health status, but also have an impact on their school adaptability [43, 44]. From the perspective of family function theory, the family system plays a core role in stress coping and resource supply. In a family environment full of conflicts, a tense family atmosphere may lead to a reduction in attention paid to children, hinder teenagers from relieving stress and affect their mental health. Perceived family conflicts may lead to a lack of emotional security, elevated levels of anxiety and fear, and a decline in mental health. Relevant research has found that students with higher levels of mental health can better abide by school rules and regulations, actively handle school affairs, and perform better in school adaptation [45]. Furthermore, a higher level of school adaptation can promote positive emotional experiences. Positive emotions act as a buffer and protection when people go through crisis events, help shape good mental health [46, 47]. According to the theory of resource conservation, the negative emotions and stress associated with perceived family conflicts consume excessive personal resources, which may reduce the resources needed for mental health and school adaptation development. Internet addiction and other unhealthy lifestyle behaviors that bring satisfaction have also been proven to be effective ways to restore personal resources [48]. Based on this, we believe that mental health and school adaptation play a chain mediating role between the perception of family conflict and unhealthy lifestyle behaviors.

Based on the above analysis, this study takes the Chinese adolescent group as the research object, by integrating multi-path mediation mechanisms, combining the Chinese cultural background, and introducing physical and digital risk behavior indicators, a chain mediation model is systematically constructed to explore the psychological pathways by which family conflict perception affects unhealthy lifestyle behaviors, aiming to provide empirical evidence and new ideas for localized intervention practices.

In conclusion, this study puts forward the following research hypotheses:

1. Perception of family conflict is associated with the unhealthy lifestyle behaviors among teenagers.
2. Mental health plays a mediating role between the perception of family conflicts and unhealthy lifestyle behaviors among teenagers.
3. School adaptation plays a mediating role between the perception of family conflicts and unhealthy lifestyle behaviors.
4. Mental health and school adaptation play a chain-like mediating role between the perception of family conflict and unhealthy lifestyle behaviors among adolescents.

**Fig. 1:**
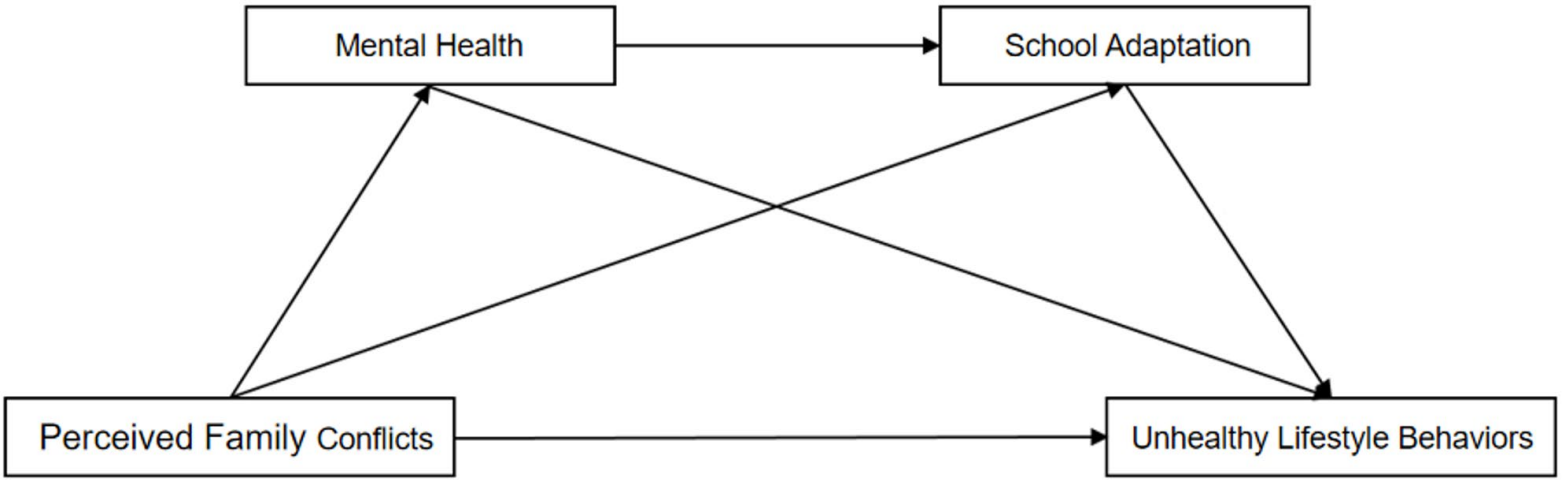
Theoretical model diagram

Based on the above three theoretical assumptions, this study constructed a theoretical assumption model, as shown in Fig. 1.

## Participants and procedure

### Participants

The data used in this study are from the Adolescent Health Thematic Database of the National Population Health Science Data Archive Center (PHDA) in China [49]. This is the first publicly available dataset in China dedicated to the health and health-related behaviors of teenagers. The data used in this study is derived from a survey conducted in Shandong Province, China from 2020 to 2021. Using the PPS sampling method, 100 schools were randomly selected from 10 administrative regions based on the specific geographical location, population and socio-economic status of the province. Schools with less than 100 students in each grade or less than 300 students in total do not meet the conditions of this study. All investigations follow the principles of informed consent, anonymity and confidentiality. The collected data include the personal information, socioeconomic status, social interaction, school adaptation, risky behavior and mental health of teenagers. This study selected a group of high school teenagers as the research subjects, excluding individuals with inconsistent responses, those missing items, and those with extremely high scores. The final sample size of this study was 9,114 people, including 4,153 males and 4,961 females. This study was approved by the Ethics Committee of Shandong University in China.

### Variables

#### Perception of family conflict

This study adopted three items from the “Family Relationship” module of the PHDA Adolescent Health Theme Database to construct a composite index of family conflict perception. The design of this measurement is based on the family function theory to comprehensively capture the different levels of family conflicts. The questions are respectively “father’s frequent getting drunk(reflecting family tension caused by unhealthy lifestyle)”, “parents often quarrel. (reflecting obvious interpersonal conflicts)” and “parents’ relationship is harmonious (reflecting the overall emotional atmosphere, adopt the negative scoring method)”. All entries should be answered in a two-point format. 1 points will be awarded for a “yes” answer and 2 point for a “No” answer. The scores of the three items are added up to form a composite indicator. The higher the total score, the lower the level of adolescents’ perception of parental conflict.The Cronbach’s coefficient for this study is 0.650.

#### Mental health

The mental health of the high school students was measured by the Chinese version of the Symptom Checklist 90 (SCL-90). The SCL-90 consists of 90 items, each scored on a scale of distress from “not at all” (0) to “extremely severe” [4], and quantifies psychopathology in terms of nine major symptoms: somatization, obsessive-compulsive symptoms, interpersonal sensitivity, depression, anxiety, hostility, phobias, paranoia and psychoticism, and sleep and its development. The higher the score, the more pronounced the subject’s self-perceived above symptoms are said to be. The reliability and validity of the Chinese version have been tested [50, 51]. The Cronbach’s alpha was 0.985 in this study.

#### School adaptation

The School Social Behavior Scales - Second Ed(SSBS-2) was published by Assessment-Intervention Resources, which is composed of two scales, Social Competence and Antisocial Competence, for screening and assessing social competence and antisocial behavior in students in grades one through twelve, and each scale consists of 32 items on a 5-point Likert-type scale. The present study used the Social Competence Scale of the SSBS-2 to measure adolescents’ adaptive school behaviors comprising three dimensions—peer relationships, self-management, and learning behaviors—with higher scores indicating better school adaptation [52, 53]. The Cronbach’s alpha was 0.968 in this study.

#### Unhealthy lifestyle behaviors

We take into account that in the current social and cultural background of China, smoking, drinking and excessive screen time are highly relevant and representative. Smoking and drinking are often regarded as symbols of adult identity, through which teenagers may seek peer recognition or express independence. Excessive screen time is a new health risk faced by modern teenagers. We evaluate the specific manifestations of unhealthy lifestyle behaviors among teenagers from the perspectives of substance use risk behaviors and digital media use risk behaviors. The issues related to risky behaviors in substance use cover both smoking and drinking. Smoking-related questions include “How old were you when you first smoked, even if you only smoked one or two puffs?” How many days have you smoked in the past 30 days? In the past 30 days, during the days when you smoked, how many cigarettes did you smoke every day? Questions related to alcohol consumption include “How many days in your life have you had at least one glass of alcohol?” When was the first time you really had a glass of wine instead of a few sips? In the past 30 days, how many days have you had at least one glass of wine? These problems reflect the use of harmful substances by teenagers. Questions about risky behaviors in the use of digital media include: “How much time do you watch TV on average from Monday to Friday each day this semester?” During this semester, from Monday to Friday, how many hours do you spend each day playing video games or using the computer to do things unrelated to study (including the time spent on QQ, wechat, iPad or other social networking programs, such as sending text messages, YouTube, Facebook or other social networking programs)? These problems reflect the health issues that may arise from teenagers’ excessive reliance on electronic devices.

Based on the above issues, data on the participation of research participants in unhealthy lifestyle behaviors were obtained. Participants answered the questions in the way they thought appropriate, and the score range for each question was from 1 to 7. The sum of the scores of the eight questions is the score of unhealthy lifestyle behaviors. The higher the score, the more frequently the research participants engaged in unhealthy lifestyle behaviors.

### Statistical analysis

The data were statistically analyzed using the SPSS 26.0 software package and the IBM SPSS macro program PROCESS version 4.0. The Shapiro‒Wilk test was used for normally distributed continuous variables, differences in the data were analyzed using a t test and analysis of variance (ANOVA), and Pearson’s correlation test was used for the relationships between all variables. We analyzed the chained mediation effect using Model 6 in PROCESS version 4.0. Finally, we analyzed the significance of the chained mediation model using a bootstrap with 5000 samples and 95% confidence intervals (CIs). A value of *P* < 0.05 was considered significant.

## Results

### General characteristics of the participants

Firstly, the demographic characteristics of the samples in the study were analyzed. The total sample size included in the study was 9,114, among which 4,153 (45.6%) were male and 4,961 (54.4%) were female. More than half of the students in the sample were in boarding schools (*n* = 6,343,69.6%); In the context of this study, boarding refers to a learning mode where students need to live on campus during their study period (usually from Monday to Thursday) and return home on weekends or holidays. Most parents expect their children to achieve good grades in the middle or above of the class (*n* = 4,553,50.0%); Most adolescent families are at a medium economic level (*n* = 7,111,78.0%). The family economic situation is measured by students’ self-assessment. The measurement is carried out by students’ self-report method. This method is based on the age and educational background of the participants. Their self-judgment is relatively reliable and reflects their subjective perception of the family’s socioeconomic status. Table 1 provides more details.

**Table 1:** Demographic characteristics of the whole sample (*N* = 9114)

Variables	Category	*N*	Percentage(%)
Gender	Men	4153	45.6
	Women	4961	54.4
Boarding	Yes	6343	69.6
	No	2771	30.4
Academic expectations	Top 5 in class	1736	19.0
	Upper middle class	4553	50.0
	Class average	1150	12.6
	No special requirements	1675	18.4
Family economic	Bad	244	2.7
situation	Not good	1136	12.5
	Fair	7111	78.0
	Good	514	5.6
	Very good	109	1.2

### Differences between sample characteristics and variable scores

The results of one-way linear regression showed that there were significant differences in the effects of gender, boarding at school, parents’ academic expectations and family economic level on health risk behaviors. Among the study population, unhealthy lifestyle behaviors occurred more frequently among participants who were male, boarded at school, and had better family finances and less frequently among participants whose parents were not academically demanding. Table 2 provides more details.

**Table 2:** Univariate analysis and description of each scale

Variables	Categories	Unhealthy lifestyle behaviors	F^a^/t^b^
Gender	Men	9.16 ± 4.33	16.84***
	Women	7.79 ± 3.24	
Boarding	Yes	8.60 ± 4.01	7.80***
	No	7.97 ± 3.38	
Academic expectations	Top 5 in class	8.81 ± 4.41	15.84***
	Upper middle class	8.24 ± 3.55	
	Class average	8.88 ± 4.13	
	No special requirements	8.14 ± 3.67	
Family economic	Bad	11.03 ± 6.19	30.34***
situation	Not good	8.6 ± 3.941	
	Fair	8.19 ± 3.55	
	Good	8.75 ± 3.91	
	Very good	13.26 ± 7.02	

***p < 0.001

^a^ Statistics were estimated by ANOVA

^b^ Statistics were estimated by t-test

### Correlation analysis

Table 3 shows the average values and bivariate correlation analysis results of the perceived family conflicts, mental health, school adaptation and unhealthy lifestyle behaviors of the research subjects. The average score of perceived family conflicts was 5.85 ± 0.44 points, the average score of mental health was 133.14 ± 46.37 points, the average score of school adaptation was 124.93 ± 23.66 points, and the average score of unhealthy lifestyle behaviors was 8.41 ± 3.84 points. Unhealthy lifestyle behaviors were significantly negatively correlated with perceived family conflicts (*r*=−0.27,*P* < 0.01) and school adaptation (*r*=−0.29,*P* < 0.01), and significantly positively correlated with mental health (*r* = 0.30,*P* < 0.01).

**Table 3:** The pearson correlation analysis among research variables

Variables	1	2	3	4
Perceived Family Conflicts	1			
Mental health	−0.24**	1		
School adaptation	0.25 **	−0.33**	1	
Unhealthy Lifestyle Behaviors	−0.27**	0.30**	−0.29**	1
M	5.85	133.14	124.93	8.41
SD	0.44	46.37	23.66	3.84

**p < 0.01

### Chain mediation effects analysis

**Fig. 2:**
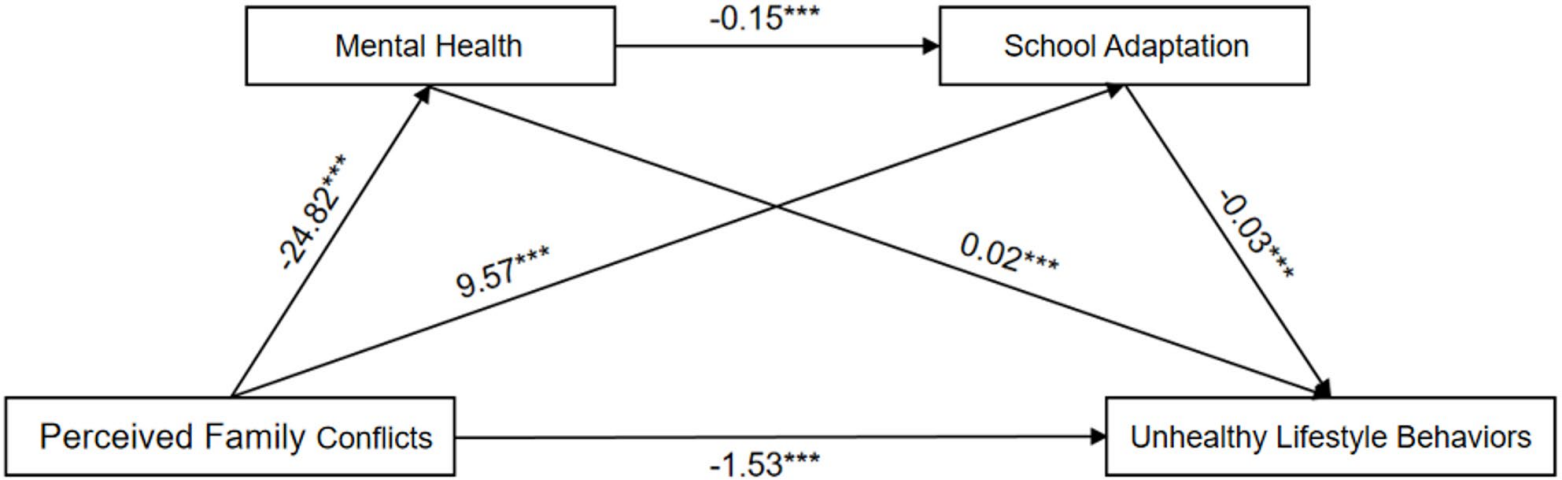
Chained mediation model diagram

Model 6 in the Process program developed by Hayes was adopted to test the mediating effect. General information such as gender, boarding, parents’ academic expectations and family economic level was controlled to analyze the mediating role of adolescent mental health and school adaptation in the relationship between family conflict perception and unhealthy lifestyle behaviors. To test the significance of the Bootstrap chained mediation, we set the number of samples to 5000, thereby generating confidence intervals to estimate the true value of the mediation effect. The results show that family conflict perception negatively predicts unhealthy lifestyle behaviors among adolescents, with an effect size of −1.53. Mental health and school adaptation significantly mediate the relationship between parental relationships and unhealthy lifestyle behaviors, with a total indirect effect size of −0.79. There are three mediating chains: (1) Indirect effect M1: Perceived family conflicts → Mental health → unhealthy lifestyle behaviors, with a value of −0.43; (2) Indirect effect M2: Perception of family conflicts → School adaptation → Unhealthy lifestyle behaviors, with an effect value of −0.26; (3) Indirect effect M3: Perception of family conflicts → Mental health → School adaptation → Unhealthy lifestyle behaviors, with an indirect effect of −0.10; The 95% ci of Bootstrap does not include 0, indicating that mental health and school adaptation have significant independent and linkage mediating effects in the perception of family conflict and unhealthy lifestyle behaviors. The specific pathways are shown in Table 4; Fig. 2.

**Table 4:** Path analysis of chained mediation effects of research variables

Pathways	Effect	Boot SE	Percentage of total effect (%)	95%CI
Perceived Family Conflicts → Unhealthy Lifestyle Behaviors	−1.53	0.13	65.95%	−1.77
				−1.27
M1: Perceived Family Conflicts → Mental Health → Unhealthy Lifestyle Behaviors	−0.43	0.04	18.53%	−0.50
				−0.36
M2: Perceived Family Conflicts → School Adaptation → Unhealthy Lifestyle Behaviors	−0.26	0.03	11.21%	−0.31
				−0.21
M3: Perceived Family Conflicts → Mental Health → School Adaptation → Unhealthy Lifestyle Behaviors	−0.10	0.01	4.31%	−0.12
				−0.08
Total mediating effect	−0.79	0.05	34.05%	−0.88
				−0.70
Total effect	−2.32	0.09	100%	−2.48
				−2.14

## Discussion

This study adopted a chain mediation model to empirically analyze the relationship between family conflict perception and unhealthy lifestyle behaviors among high school adolescents in Shandong Province. The analysis results support the pre-proposed theoretical framework.The results of this study indicate that mental health and school adaptation play a partial mediating role in the relationship between perception of family conflict and unhealthy lifestyle behaviors. Perception of family conflict affects unhealthy lifestyle behaviors through three pathways: mental health, school adaptation, and mental health→school adaptation. This will help us further understand the relationship mechanism between perceived family conflicts and unhealthy lifestyle behaviors among teenagers, and also assist schools, relevant institutions and third-party organizations in taking measures to further prevent and improve unhealthy lifestyle behaviors among teenagers.

### Analysis of differences in scores of unhealthy lifestyle behaviors

According to the results of the demographic difference analysis, the scores of unhealthy lifestyle behaviors of male participants were significantly higher than those of female participants, which was similar to the research results of Assanangkornchai [54]. Because men are usually regarded as adventurers, they are more involved in most unhealthy lifestyle behaviors and substance use than women [55]. Boarding teenagers are more likely to engage in behaviors that are harmful to health. Boarding is a closed learning and living environment. Compared with non-boarding students, teenagers have less contact with their parents and the outside world. Boarding students may have more depressive symptoms [56] and other unhealthy behaviors. However, the influence of boarding schools needs to be viewed dialectically, boarding environments may serve as a buffer zone [57]. For adolescents from families with high conflict or dysfunction, boarding at school exerting a protective effect on mental health by reducing the frequency of their direct exposure to family conflicts and indirectly lowering the risk of unhealthy behaviors resulting from this. Research shows that a supportive school environment itself is an important protective factor for the health of teenagers. Therefore, the impact of boarding education is not a single negative factor. Its ultimate effect may depend on the combined effect of multiple factors such as the quality of the original family environment and the supportive level of school management.

Teenagers whose parents pay less attention to their studies have a lower frequency of unhealthy lifestyle behaviors. For Chinese teenagers, parents are one of the important sources of academic pressure. Chinese parents usually show greater commitment and expectations for their children’s education [58]. When children fail to meet their parents’ expectations, they will be criticized and punished by their parents, which is highly related to stress. Academic pressure positively predicts depression and problem behaviors in adolescents [59, 60]. Teenagers from wealthy families are more likely to engage in unhealthy lifestyle behaviors. This might be because wealthy families tend to provide more material support for their children, and Ji’s research shows that teenagers with higher spending are more likely to belong to the high-risk group of unhealthy lifestyle behaviors [55]. However, another research shows that teenagers with a low socioeconomic status are more likely to exhibit various unhealthy lifestyle behaviors. This might be because teenagers from families with lower socioeconomic status have a lower positive attitude towards maintaining healthy behaviors, face higher levels of peer pressure, and experience more stressful life events [8]. Therefore, there is a lack of clear support to explain the relationship between socioeconomic status and unhealthy lifestyle behaviors [61].

### Perceive the impact of family conflicts on unhealthy lifestyle behaviors

Based on the chain mediation model constructed in this study, we verified that family conflict perception is a negative predictor of health risk behaviors among adolescents, and that family conflict perception among adolescents increases the risk of problem behaviors among adolescents. This finding is similar to the results of a series of studies on the influence of parental relationships on children, in which marital disorder is one of the causes of various problem behaviors in children [62–64]. Some scholars have even proposed that where there are problem children, there are problem marriages [65]. Teenagers with a high perception of family conflicts lack attention and emotional support. They might hope to gain their parents’ attention by engaging in behaviors that do not meet the expectations of their families and society, such as smoking and drinking, or by satisfying their social needs through mobile games and social networking software. Family conflict perception and psychological distres have exacerbated mobile phone addiction behavior [66, 67]. Therefore, perceived family conflicts may have a non-negligible impact on children. Since the perception of family conflicts is inevitable, Cummings believes that transforming conflicts into constructive ones can enhance children’s emotional security [68]. Furthermore, Barthassat believes that children from families with constructive conflicts have good emotional regulation abilities [69]. These abilities may understand others’ emotions and generate positive social behaviors, and reduce the frequency of unhealthy lifestyle behaviors. Furthermore, the spillover hypothesis of family systems theory holds that couples who have harmonious relationships and receive satisfaction and support from each other are more sensitive to many of their children’s needs [70, 71], and their children have a higher level of emotional security. However, some scholars do not fully agree with the above view; Not all teenagers will develop unhealthy lifestyle behaviors due to perceived family conflicts. Even within the same family environment, the influence of parents’ behaviors on their children may vary depending on the situation or the quality of the relationship [72]. The research results also indicate that some children exposed to families with poor parental relationships and high conflict frequencies do not exhibit serious problem behaviors [73]. Besides constructive conflict handling and emotional security at the family level, psychological traits formed by individuals during development, such as self-regulation ability and psychological resilience, also play a key role. Good self-regulation ability helps teenagers suppress impulsive behaviors and manage goal-oriented behaviors when facing family pressure, and reduces the risk of their unhealthy behaviors [74]; Teenagers’ active participation in sports is significantly associated with a higher level of resilience [75], which may become an important buffer path to alleviate the negative impact of family stress.

Overall, the results of this study verify the hypothesis that family conflict perception predicts the occurrence of unhealthy lifestyle behaviors among adolescents. However, since the perception of unhealthy lifestyle behaviors resulting from family conflicts is a multi-factor approach and some the previous research results differ from ours, the next consideration is to expand the sample size and increase the research hypotheses.

### The mediating role of mental health

In this study, we verified the partial mediating role of mental health in perceiving family conflicts and unhealthy lifestyle behaviors. That is to say, we have found that teenagers who perceive a high level of family conflict have a lower level of mental health and are more likely to engage in unhealthy lifestyle behaviors. Family conflicts largely affects the harmony and happiness of the family, as well as the psychology and behavior of children [76]. Studies have shown that the perception of family conflicts indirectly affects the mental health of teenagers by influencing the quality of parent-child relationships [77]. Marital conflicts may be related to bad relationship between parents and teenagers, ultimately affecting the mental health of teenagers [78]. Negative family events related to the perception of family conflicts may place adolescents in an unstable and unsafe family atmosphere [79]. It may trigger negative emotions such as depression.Studies show that young children who witness these negative family events are more likely to develop discipline issues, mood swings, and difficulty leaving parents [80]. For a long time, teenagers have been exposed to their parents’ negative emotions and behaviors, constantly feeling the tense atmosphere in the family. When confronted with problems, they tend to display more negative emotions and withdrawal behaviors, thus forming negative coping styles. Wang’s research also indicates that middle school students’ experiences of negative life events are risk factors for Internet addiction [81]. Furthermore, other studies have shown that an individual’s addictive behavior is due to excessive exposure to stressful events in life, which potentially leads to a decline in the satisfaction of their psychological needs and seeks compensation and relief of emotional pain through addictive activities [82, 83]. In conclusion, teenagers’ perception of family conflicts and the tense atmosphere within them are highly correlated with their own negative emotions, and their psychological needs are not met, which is not conducive to the development of their mental health. The lower the satisfaction with psychological needs, the more likely an individual is to engage in behaviors that are harmful to health.

### The mediating role of school adaptation

In addition, this study validated the mediating role of school adaptation between perceived parental conflict and adolescent health risk behaviors. The results show that adolescents’ perceptions of parental conflict make them more prone to health risk behaviors by showing maladaptive situations in school and problems in academic performance and peer relationships. First, children learn from observing their parents about dealing with relationships, and the way children perceive and respond to their parents’ conflicts may develop into aggressive interaction patterns, which are not conducive to the development of peer interaction and good school adaptability among teenagers [84]. In addition, a good parental marital relationship provides emotional support and a sense of security, which may contribute to adolescent school adaptation development [85]. Tense or conflicting marital relationships between parents can be relevant to internal problems such as depression in teenagers [86], which may affect their adaptation to school.Maladaptation at school may cause adolescents to have a negative attitude toward their future academic life, become reluctant to interact with classmates, unwilling to participate in social activities, feelings of loneliness and alienation, a decreased sense of presence in the group and gradual marginalization (peer pressure, decreased sense of self-worth), and ultimately, the emergence of health risk behaviors [87]. Therefore, perceived parental conflict can be detrimental to the development of school adaptation in adolescents, and additional measures are necessary to help adolescents adjust to school and ultimately reduce the risk of developing health risk behaviors.

### Chain mediation of school adaptation and mental health

This study found that there is a close connection between mental health and school adaptation. The two constitute the intermediate link of perceiving family conflicts → mental health → school adaptation → the impact of unhealthy lifestyle behaviors, and play a chain mediating role in perceiving the impact of family conflicts on unhealthy lifestyle behaviors. In other words, teenagers who experience more perception of family conflicts have lower mental health levels, which makes it more difficult for them to adapt to school life and more prone to unhealthy lifestyle behaviors. As guardians of teenagers, parents provide them with security guarantees, which are related to teenagers’ good emotional regulation [88].And emotional security is also a key factor influencing an individual’s interpersonal behavior and social activities [89]. On the other hand, destructive family conflicts undermine the safety and security of teenagers.Teenagers feel threatened about their emotional safety in a family conflict environment, which may be related to maladaptive emotional and behavioral responses as well as mental health problems [90].Meanwhile, conflicts among families increase the possibility that teenagers will show stress responses and reduce their potential to achieve excellent academic performance [91]. When their academic performance is poor, teenagers are less likely to believe that they can complete their academic tasks and adapt to school life [92]. Furthermore, studies have shown that there is a positive correlation between mental health and school adaptation [45]. Teenagers with poor mental health perform poorly in school adaptation indicators. This may lead to estrangement from the school’s peer group, identification with unconventional or deviated peer groups, and participation in deviated activities and unhealthy lifestyle behaviors [93, 94].Moreover, unhealthy lifestyle behaviors can be regarded as self-protective actions of individuals to alleviate anxiety and depression symptoms caused by low-level mental health and stressful life events [83]. Overall, due to having experienced family conflicts in their original families, teenagers are more likely to engage in behaviors that are harmful to their health, resulting in lower mental health levels and subsequently poor performance in school adaptation indicators. Therefore, when preventing unhealthy lifestyle behaviors among teenagers, it should be taken into account that the ultimate goal can be achieved by improving mental health levels and school adaptation.

## Limitations

This study has several limitations. Firstly, the samples of this study are from Shandong Province, China. Shandong Province is a part of eastern China and does not represent the whole teenagers. Therefore, our research results have limitations in terms of sample representativeness and cross-cultural applicability. Future research we will expand the sample coverage to improve the representativeness and generalizability of the study. Secondly, the variables of this study are derived from self-reports of adolescents, which may be influenced by recall bias. Thirdly, although this study discussed the mediating relationship among variables, adolescent mental health is a multi-dimensional issue. Subsequent research can conduct in-depth analysis of the relationship between the internal dimensions of mental health and unhealthy lifestyle behaviors, incorporating more complex variables.

## Conclusions

This study took 9,114 high school students in Shandong Province as samples, integrated the theory of emotional safety and the theory of resource conservation, and for the first time constructed and verified a chain multi-mediation model, systematically revealing the complete action path of “perception of family conflict →mental health→school adaptation→unhealthy lifestyle behaviors”. It has been made clear that mental health and school adaptation are the core intervention targets for preventing the spillover of family conflict perception to unhealthy lifestyle behaviors. To effectively prevent the transmission of family conflict perception to teenagers’ unhealthy lifestyle behaviors, we suggest: (1) At the family level, parents should create a stable, supportive and loving environment, resolve conflicts through calm, positive communication, mutual understanding and compromise, and minimize their negative impact to the greatest extent; (2) At the school level, educators should enhance students’ psychological resilience and school adaptability simultaneously through mental health courses, academic support, peer assistance and diverse campus activities, and reduce their sensitivity to family conflicts (3) At the policy level, integrate resources from families, schools, communities and doctors, build a hierarchical intervention and referral network, provide continuous attention and resource support for teenagers in need, help them actively cope with external pressure, and ultimately reduce the occurrence of unhealthy lifestyle behaviors.Future research can be based on the findings of this study to design and evaluate multi-level intervention programs targeting families, schools and communities, and verify their effectiveness in improving adolescent mental health, school adaptation and reducing unhealthy behaviors through experimental studies.

## Data Availability

The data sets used and or analyzed during this study are available from the corresponding authers upon reasonable request.
